# A Friendly Relationship between Endophytic Fungi and Medicinal Plants: A Systematic Review

**DOI:** 10.3389/fmicb.2016.00906

**Published:** 2016-06-09

**Authors:** Min Jia, Ling Chen, Hai-Liang Xin, Cheng-Jian Zheng, Khalid Rahman, Ting Han, Lu-Ping Qin

**Affiliations:** ^1^Department of Pharmacognosy, School of Pharmacy, Second Military Medical UniversityShanghai, China; ^2^Department of Physiological Biochemistry, School of Pharmacy and Biomolecular Sciences, Liverpool John Moores UniversityLiverpool, UK

**Keywords:** endophytic fungi, medicinal plant, population structure, plant-microbe interaction, secondary metabolite

## Abstract

Endophytic fungi or endophytes exist widely inside the healthy tissues of living plants, and are important components of plant micro-ecosystems. Over the long period of evolution, some co-existing endophytes and their host plants have established a special relationship with one and another, which can significantly influence the formation of metabolic products in plants, then affect quality and quantity of crude drugs derived from medicinal plants. This paper will focus on the increasing knowledge of relationships between endophytic fungi and medicinal plants through reviewing of published research data obtained from the last 30 years. The analytical results indicate that the distribution and population structure of endophytes can be considerably affected by factors, such as the genetic background, age, and environmental conditions of their hosts. On the other hand, the endophytic fungi can also confer profound impacts on their host plants by enhancing their growth, increasing their fitness, strengthening their tolerances to abiotic and biotic stresses, and promoting their accumulation of secondary metabolites. All the changes are very important for the production of bioactive components in their hosts. Hence, it is essential to understand such relationships between endophytic fungi and their host medicinal plants. Such knowledge can be well exploited and applied for the production of better and more drugs from medicinal plants.

## Introduction

It is widely considered in a conventional view that the quality and quantity of crude drugs originated from medicinal plants are largely affected by such factors as the genetic background of the concerned plants, ecological habitats where the plants live, and soil nutrients (Dai et al., 2003; Sherameti et al., 2005). However, in the recent years, it is gradually recognized that endophytic fungi or endophytes have played a very important role in affecting the quality and quantity of the crude drugs through a particular fungus-host interaction, indicating that more understanding on the particular relationships between endophytic fungi and medicinal plants is required for promoting crude drug production (Faeth and Fagan, 2002). Although endophytic fungi are one of the most important elements in plant micro-ecosystems that should have significant influences on the growth and development of host plants, our knowledge about the exact relationships between endophytic fungi and their host plants is still very limited. Understanding and exploiting such relationships will facilitate the ideal production of better drugs by manipulating the growth conditions of medicinal plants by, for example, adding a particular group of endophytic fungi to the plants to improve the drug quality and quantity (Firáková et al., 2007). Ideally, an alternative method can be developed to directly produce desired drugs through bioengineering of the selected medicinal plants and endophytic fungi under a certain cultural condition, if the fungus-host relationships and their metabolic mechanisms under cultural conditions are well understood (Kumaran et al., 2008, 2009). Such an industry style of manufacture may replace the traditional way to produce drugs, which essentially depends on natural medicinal plants.

Endophytic fungi belong to mitosporic and meiosporic ascomycetes that “asymptomatically reside in the internal tissues of plants beneath the epidermal cell layer, where they colonize healthy and living tissue via quiescent infections” (Bacon and White, 2000). There is a great biological diversity of endophytic fungi, occurring naturally in the temperate regions and tropical rainforests, where about 300,000 terrestrial host-plant species are distributed. Each plant species hosts one or more endophytic fungus species. Endophytic fungi are diverse polyphyletic groups of microorganisms, and can thrive asymptomatically in different healthy tissues of living plants above and/or under the ground, including stems, leaves, and/or roots. It is estimated that over one million endophytic fungal species occurring in the nature (Faeth and Fagan, 2002). Schultz classified the fungal endophytic fungi into three main ecological groups: (a) mycorrizal; (b) balansicaeous or pasture endophytic fungi; and (c) non pasture endophytic fungi (Faeth and Fagan, 2002). The bioactive compounds produced by endophytic fungi, exclusive of those to their host plants, are very important to increase the adaptability of both endophytic fungi and their host plants, such as the tolerances to biotic and abiotic stresses. In addition, these compounds can induce the production of a plethora of known and novel biologically active secondary metabolites (Zhang et al., 2006; Firáková et al., 2007; Rodriguez et al., 2009) that can be exploited and applied by human as important medicinal resources.

It is known that the colonization of endophytic fungi is not an incidental opportunity because of the chemotaxis that is specific chemicals produced by the host plants. At the same time, different types of secondary metabolites, such as saponin and essential oils from medicinal plants, are produced through long-term co-evolution as a resistance mechanism to the pathogens, most possibly including endophytic fungi. Therefore, the secondary metabolites became obstacles for the colonization of endophytic fungi. To overcome this, endophytic fungi must secrete the matching detoxification enzymes, such as cellulases, lactase, xylanase, and protease, to decompose these secondary metabolites before they penetrate through the defense systems of the resided host-plants. Once inside the tissues of a host-plant, the endophytic fungi assumed a quiescent (latent) state, either for the whole lifetime of the host plant (neutralism) or for an extended period of time (mutualism or antagonism) until environmental conditions are favorable for endophytic fungi or the ontogenetic state of the host changes to the advantage of the fungi (Sieber, 2007).

During the long period of co-existence and evolutionary processes, different relationships have been established between endophytic fungi and their host plants through a particular fungus-host interaction recognized as: (i) a continuum of mutualism, (ii) antagonism, and (iii) neutralism. The genetic background, nutrient level, and ecological habitats of the medicinal host plants are considered as the pressure-choice factors on the population structure of the endophytic fungi that, in turn, confer some kinds of benefits, such as the induced growth, increased resistance to disease, and/or herbivore (Rodriguez et al., 2009), as well as accumulated bioactive components (Firáková et al., 2007), some of which can be used by human as beneficial medicines. Therefore, the mutual interrelation between endophytic fungi and their host plants can impose certain effects on the formulation of some types of bioactive compounds that can be used by human.

In this paper, we reviewed the studies of endophytic fungi and medicinal plants for the last 30 years, with a particular emphasis on the factors that possibly influence the population structure and distribution of endophytic fungi and benefits to their host plants from the existence of endophytic fungi. We hope that this review will provide readers useful information for understanding the environmental and host-plant factors affecting endophytic fungi as well as the friendly relationships between endophytic fungi and medicinal plants, which may help researchers make better use of the beneficial symbiosis and expand the way for obtaining high-quality resources of certain medicinal plants. Ideally, a system mimicking the mutualistic or antagonistic symbiosis conditions of endophytic fungi and their host plants may be established to effectively produce the desired drug compounds through bioengineering, if the relationships and conditions that promote the production of the compounds are clearly understood. This review will also discuss the existing problems in research and potential applications of endophytic fungi for drug production.

## Environmental and host-plant factors affecting endophytic fungi

**Figure 1:**
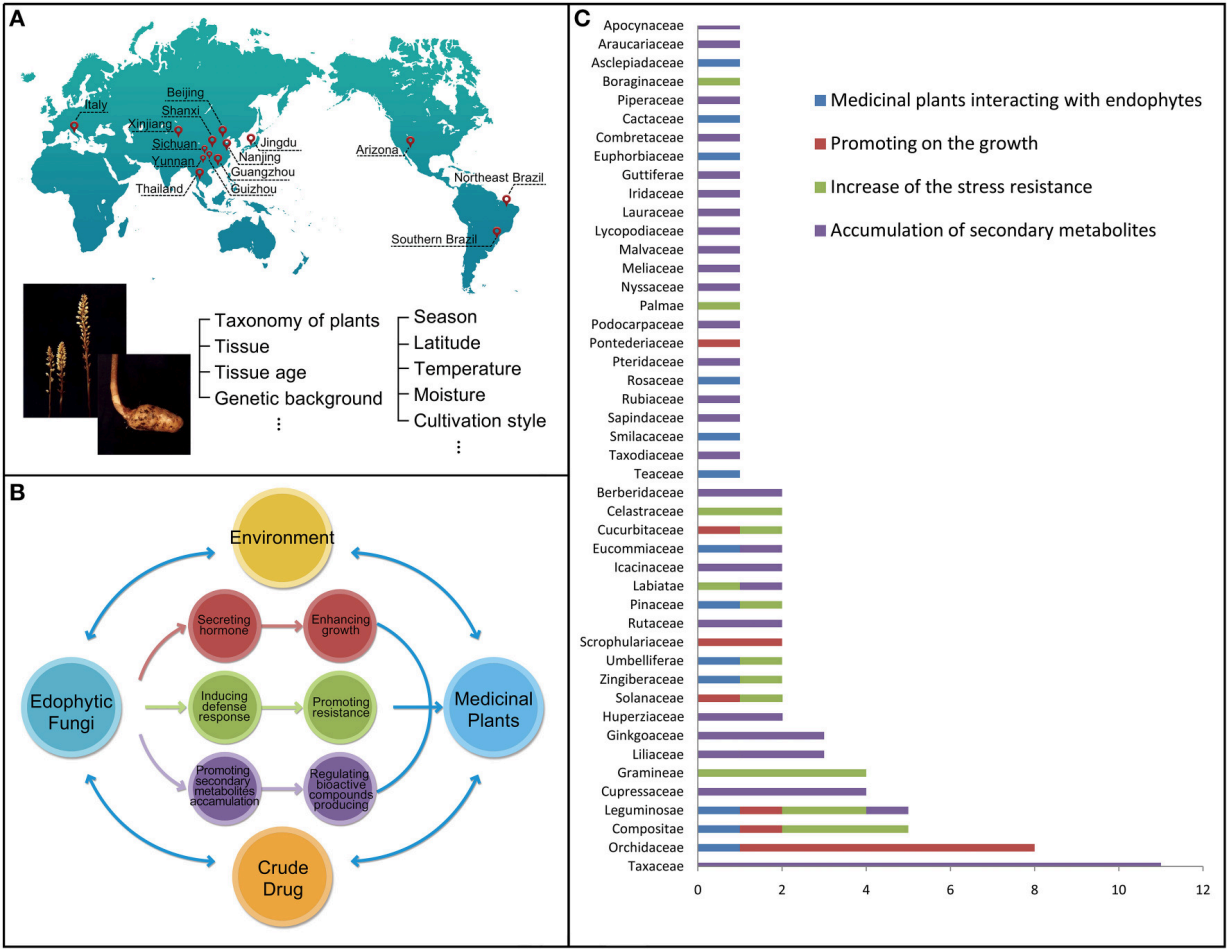
**The host-plant and environmental factors affecting the population structure and distribution of endophytic fungi (A)**. The beneficial relationships established by the endophytic fungi to their host medicinal plants including enhancing the growth and resistance of their host plants, as well as promoting the accumulation of secondary metabolites **(B)**. Taxonomy of the total of 96 medicinal plant species involved in the reference survey and analysis for last 30 years (x axis: species numbers of the family; y axis: the type of the family) **(C)**.

Results of the analyses also indicated that the population structure or distribution pattern of endophytic fungi was significantly associated with the variation in environments, as well as the classification and genetic background of host plants (Table 1, Figure 1A). Data from the reference analysis suggested that some environmental conditions, such as temperature, humidity, illumination, geographic location, and vegetation significantly affected the distribution pattern of endophytic fungi (Suryanarayanan et al., 2005; Song et al., 2007). For example, particular conditions determined the distribution ranges of host plants that in return determine the species of endophytic fungi and their spore germination, growth, reproduction, and metabolism during the entire life cycle. Similarly, results from the analyses suggested that the distribution of certain endophytic fungal populations was only restricted to particular host plant species (or families) and particular genetic background (genotypes) of a species (Dai et al., 2003; D'Amico et al., 2008). This finding is particularly important because the non-random distribution of endophytic fungi will determine the production of diverse secondary metabolites promoted by endophytic fungi that can be used by human as drugs. In addition, the secondary metabolites may confer different benefits to the host plants, such as enhancing the growth and resistance to biotic and abiotic stresses, which provides opportunities for us to understand the relationships between endophytic fungi and medicinal plants. Below, we would present the influences of ecological environments and genetic background/tissues of host plants on the population structure of endophytic fungi, respectively.

**Table 1:** **Influences of host medicinal plants on the population structure of endophytic fungi**.

**Family of host plants (represent species)**	**Isolation part**	**Habitat**	**Factor affecting the population structure**	**References**
Cactaceae (*Cactus* sp.)	Stem	Desert of tropical savanna	Environment: moisture^a^ and temperature^b^	Suryanarayanan et al., 2005
Rosaceae (*Malus domestica*)	Leaf, flower, fruit	Tropical rainy region	Environment: cultivation style^c^	Camatti-Sartori et al., 2005
*Leguminosae (Glycyrrhiza inflat*)	Root	Salinized sandy land in warm temperate region	Environment: moisture^a^ and temperature^b^	Song et al., 2007
Eucommiaceae (*Eucommia ulmoides*)	Leaf, branch, bark	Subtropical mountain and warm temperate semi-humid region	Environment: latitude^e^ and temperature^b^	Sun J. et al., 2008
			Tissue^d^	
Orchidaceae (*Gastrodia elata*)	Tuber, flower	Hillside forests, wetland in temperate plateau	Enviroment: latitude^e^	Mo et al., 2008
			Tissue^d^	
Euphorbiaceae (*Sapium sebiferum*)	Leaf, twig	Mountain in subtropics	Genetic background^f^	Dai et al., 2003
			Tissue^d^	
Smilacaceae (*Heterosmilax japonica*)	Stem	Subtropical monsoon region	Season^g^	Gao et al., 2006
Pinaceae (*Pinus tabulaeformis*)	Bark, needle, xylem	Forests in warm temperate semi-humid monsoon region	Season^g^	Guo et al., 2008
			Tissue age^h^	
Teaceae (*Camellia japonica*)	Leaf	Temperate secondary forest	Season^g^	Osono, 2008
			Tissue age^h^	
Umbelliferae (*Apium graveolens, Cichorium intybus, Foeniculum vulgare, Lactuca sativa*)	Leaf, root, seed	Mediterranean region	Taxonomy of plants^f^	D'Amico et al., 2008
Zingiberaceae (*Amomum siamense*)	Leaf, pseudostem, rhizome	Tropical monsoon forest	Tissue^d^	Bussaban et al., 2001
Compositae (*Atractylodes lancea*)	Rhizome	Mountain in subtropics	Tissue^d^ and age of tissue^h^	Wang Y. et al., 2009
Asclepiadaceae (*Calotropis procera*)	Leaf	Garden bed	Tissue^d^	Nascimento et al., 2015

a *The endophyte colonization was positively correlated with humidity*.

b *The lower species diversity of the endophyte in temperate plants than that in tropical forests trees*.

c *The highest endophytes number under organic cultivation*.

d *The colonization rates of endophytic fungi from high to low in different tissues were bark>needle>xylem*.

e *Different dominant endophytic fungi*.

f *Specific host–endophyte combinations*.

g *The colonization rates of endophytic fungi from high to low were spring>winter>autumn>summer*.

h *The species richness of endophytic fungi increased as tissue aged, especially leaves*.

### Influences of ecological environments on population structure of endophytic fungi

We found that ecological or environmental conditions, such as temperature, humidity, and levels of soil nutrition were important factors to determine the types and amount of secondary metabolites of the host plants, which would indirectly affect the population structure of the endophytic fungi. For example, under the conditions of low mean annual sunshine hour and the high mean annual humidity, the host medicinal plants would produce more nutrients that were suitable for the colonization, reproduction, and dissemination of the endophytic fungi (Wu et al., 2013). In contrast, under the cold climatic conditions and inappropriate rates of respiration, oxygen concentration, and pH value, only certain types of host species could successfully grown. As a consequence, only a limited number of particular endophytic fungi could colonize in the corresponding host plants, resulting a certain degree of regional specificity on population structure of endophytic fungi (Jiang et al., 2010).

We also found that population structure of endophytic fungi normally represented a certain degree of regional specificity. The distribution of endophytic fungi from the same regions presented a high degree of similarity in terms of species taxonomy (D'Amico et al., 2008). Conversely, species and their population structure of endophytic fungi even in the same host plant species from different regions normally presented very low degree of similarity (Jiang et al., 2010).

### Influences of genetic background of host medicinal plants on population structure of endophytic fungi

The analysis of relationships between the host genotypes and symbiotic lifestyle expression further revealed that individual isolates of some endophytic fungal species could express either parasitic or mutualistic lifestyles, depending on the colonized host genotype (Redman et al., 2001; Unterseher and Schnittler, 2010). Accordingly, the fungus-host plant relationships should be regarded as flexible interaction, whose directionality was determined by slight differences in fungal gene expression in response to the host reaction, or conversely, by host recognition and response to the fungi. Hence, slight genetic differences in the genomes of both partners controlled the outcome (positive, negative, or neutral) of the symbiosis (Moricca and Ragazzi, 2008). Thus, population structure of endophytic fungi was considerably affected by the genetic background of host plants. Based on the facts indicated by the analyzed references that the fitness of the endophytic fungi largely depended on the fitness of the host medicinal plants, suggesting that the host plants largely determined the colonization and distribution of endophytic fungi in the host plants (Saikkonen et al., 2004).

Furthermore, phase disposition (age) of host plants and tissues may likewise influence species composition of the endophytic community (Sieber, 2007). For example, different endophyte species were found in different tissues such as parenquima, vascular ducts, and dermis of a host plant with different ages (Rodrigues, 1994). Such a specific distribution of endophytic species might be related to their ability to utilize specific substrates (Rodrigues, 1994). In addition, differential substrates utilized by different endophytic species demonstrated their resource distribution strategy when lived in the same organ of a host (Carroll and Petrini, 1983), reducing the competition between the endosymbionts. This indicated that the colonization of endophytic fungi was significantly determined by different plant tissues producing differential substrates.

## Beneficial relationships conferred by endophytic fungi to host plants

Our analysis based on the selected references further indicated the benefits conferred by some endophytic fungi to their host plants after colonization. Such a beneficial interaction could be presented from three different aspects (Figure 1B). First, some endophytic fungi could produce different plant hormones to enhance the growth of their host plants (Waqas et al., 2012). For example, the growth of wheat (*Triticum aestivum* L.) could be enhanced by *Azospirillum* sp. under drought stresses (Dingle and McGee, 2003). Second, some endophytic fungi would produce different bioactive compounds, such as alkaloids, diterpenes, flavonoids, and isoflavonoids, to increase the resistance to biotic and abiotic stresses of their host plants (Firáková et al., 2007; Rodriguez et al., 2009). Third, some endophytic fungi could promote the accumulation of secondary metabolites (including important medicinal components or drugs) originally produced by plants. These metabolites may be produced by both of the host plants or/and endophytic fungi according to the references surveyed (Shwab and Keller, 2008). Owning to the importance of the three aspects, we would present the three types of possible beneficial endophytic fungus-host relationships accordingly.

### Classification of host medicinal plants interacting with endophytic fungi

The reference survey and analysis showed that a total of 96 medicinal plant species were mutualisms, meaning mutual benefits, in terms of the fungus-host relationships (Tables 1–**4**). These species were distributed among 46 families (Figure 1C), including Apocynaceae (1 taxon), Araucariaceae (1 taxon), Asclepiadaceae (1 taxon), Berberidaceae (2 taxa), Boraginaceae (1 taxon), Cactaceae (1 taxon), Celastraceae (2 taxa), Combretaceae (1 taxon), Compositae (5 taxa), Cucurbitaceae (2 taxa), Cupressaceae (4 taxa), Eucommiaceae (2 taxa), Euphorbiaceae (1 taxon), Ginkgoaceae (3 taxa), Gramineae (4 taxa), Guttiferae (1 taxon), Huperziaceae (3 taxa), Icacinaceae (2 taxa), Iridaceae (1 taxon), Labiatae (2 taxa), Lauraceae (1 taxon), Leguminosae (5 taxa), Liliaceae (3 taxa), Lycopodiaceae (1 taxon), Malvaceae (1 taxon), Meliaceae (1 taxon), Nyssaceae (1 taxon), Orchidaceae (8 taxa), Palmae (1 taxon), Pinaceae (2 taxa), Piperaceae (1 taxon), Podocarpaceae (1 taxon), Pontederiaceae (1 taxon), Pteridaceae (1 taxon), Rosaceae (1 taxon), Rubiaceae (1 taxon), Rutaceae (2 taxa), Sapindaceae (1 taxon), Scrophulariaceae (2 taxa), Smilacaceae (1 taxon), Solanaceae (3 taxa), Taxaceae (11 taxa), Taxodiaceae (1 taxon), Teaceae (1 taxon), Umbelliferae (2 taxa), and Zingiberaceae (2 taxa). The included plant species are commonly used as medicine either by direct consumption or for extracting bioactive components.

Obviously, these medicinal plant species from different families have their distribution in particular ecological habitats. Among these species, 16 species, such as *Glycyrrhiza uralensis, Phellodendron amurense*, and *Rehmannia glutinosa* etc. were mainly distributed in temperate regions, and 20 species, such as *Amomum siamense, Cinchona ledgeriana*, and *Cinnamomum camphora* chvar. Borneol etc. were only found in tropical regions. Forty species, such as *Atractylodes lancea, Dysosma veitchii*, and *Salvia miltiorrhiza* etc. were mainly distributed in subtropical regions. Four species, such as *Apium graveolens* and *Foeniculum vulgare* etc. were mainly distributed in Mediterranean region. Interestingly, some species were only found in extreme conditions, such as *Cactus* sp. in savanna deserts, *Saussurea involucrata, Sinopodophyllum hexandrum*, and *Pedicularis* sp. in high elevation.

The data obtained from the taxonomy of the total medicinal plants involved in the reference survey and analysis for last 30 years (Figure 1C) showed that the species associated with Taxaceae and Orchidaceae are higher than that of other family and accounted for 11 and 8, respectively.

Among the plants of family Taxaceae, all are related with endophytes which can produce taxol with antitumor activity. In 1993, an endophytic fungus, *Taxomyces andreanae*, was isolated from the bark of *Taxus brevifolia* and was shown to produce Taxol under *in vitro* axenic culture conditions (Stierle et al., 1993). Numerous reports are available on the pronounced variability in Taxol production from various endophytic fungal isolates across different batch cultures (Gangadevi and Muthumary, 2009). Paclitaxel (taxol) is a kind of diterpenoids American scientists isolated from the Pacific yew extract as a natural secondary metabolites in 1960s. It has significant anti-tumor activity, particularly ovarian cancer, uterine cancer, breast cancer with high incidence. So these important discoveries are worth further studying. Followed by the family Taxaceae, papers reporting Orchidaceae accounted of eight for the second highest reports and it has the potential to be developed further. Most of them are related with the endophytes which can promote on the growth of the host plants (Zhang J. et al., 1999; Guo and Wang, 2001). In nature, almost all orchid endophytic fungi invariably belong to the genus *Rhizoctonia* and are believed to be essential symbionts for both the germination of seeds and the development of the young heterotrophic plantlets. In most orchids the plant eventually becomes photosynthetic, while some species are known to remain heterotrophic throughout their life for providing nutrition to survive. The endophyte found in the adult plant is generally assumed to be the true symbiont of seeds and protocorms, and from the behavior of endophytes isolated from roots in culture with the host seeds various views have been put forward about specificity of the relationship between hosts and endophytes. Thus, it is of great importance to study the relationship between orchids and their endophytic fungi, as well as the plants of these two families.

### Promotion of fitness and growth of host plants

Results indicated that some endophytic fungi could increase the fitness and growth of host plants by increasing hormones, such as indole-3-acetic acid, indole-3-acetonitrile, and cytokinins. Endophytic fungi could also promote the growth of their host plants by obtaining nutritional elements such as nitrogen and phosphorus useful for plants (Zhang et al., 2006; Hartley and Gange, 2009). For example, *Mycena dendrobii* could promote the seed germination and growth of the host plant *Gastrodia elata* by secreting indoleacetic acid (Guo and Wang, 2001). In addition, *Metarhizium robertsii* translocated nitrogen directly from insects to its host plants through hyphae (Behie et al., 2012). Interestingly, results showed that most hormones were produced by endophytic fungi isolated from the roots of host plants. A few references also reported that some endophytic fungi could promote the growth and fitness of the host plants by activating the expression of a certain enzymes and genes (Chen et al., 2005). For example, *Piriformospora indica* increased the growth of tobacco roots by stimulating the expression of nitrate reductase and the starch-degrading enzyme (glucan-water dikinase) (Sherameti et al., 2005) (Table 2).

**Table 2:** **Host medicinal plants with enhanced growth conferred by endophytic fungi**.

**Host plant**	**Endophytic fungi**	**Mechanism**	**References**
*Atracty lancea*	*Sclerotium* sp.	Increase cell protection from desiccationin and leaf metabolic capability of host	Chen et al., 2008
*Cucumis sativus*	*Phoma glomerata, Penicillium* sp.	Secret phytohormones viz. Gibberellins and Indoleacetic acid	Waqas et al., 2012
*Anoectochillus formosanus*	*Epulorhiza* sp.	Enhance four enzyme activities enzyme activities of chitinase, β-1, 3-glucase, phenylalanine ammonia- lyase, and polyphenoloxidase	Tang et al., 2008
*Anoectochilus roxburghii*	*Epulorhiza* sp., *Mycena anoectochila*	Enhance enzyme activities	Yu and Guo, 2000; Chen et al., 2005
*Cymbidium sinense*	*Mycena orchdicola*	Secret the plant hormones	Zhang J. et al., 1999
*Dendrobium candidum*	*Mycena dendrobii*	Secret the plant hormones	Zhang J. et al., 1999
*Dendrobium nobile, D. chrysanthum*	*Epulorhiza* sp., *Mycena* sp., Tulasnellales, Sebacinales, Cantharellales	Enhance the absorption of nutrient in plants promoting the seed germination of host	Chen and Guo, 2005
*Gastrodia elata*	*Mycena dendrobii, M. osmundicola, Mycena orchidicola, M. anoectochili*	Secret the plant hormones promoting the seed germination of host	Guo and Wang, 2001
*Pecteilis susannae*	*Epulorhiza* sp., *Fusarium* sp.	Enhance the absorption of N, P, and K elements in plants promoting the seed germination of host	Chutima et al., 2011
*Monochoria vaginalis*	*Penicillium* sp., *Aspergillus* sp.	Secret gibberellin	Ahmad et al., 2010
*Pedicularis* sp.	Dark septate endophytic fungi (DSEF)	Increase their nutrient utilization efficiency	Li and Guan, 2007
*Rehmannia glutinosa*	*Ceratobasidium* sp.	Secret indoleacetic acid	Chen B. et al., 2011
*Nicotiana attenuata*	*Sebacina vermifera*	Enhance the absorption of nutrient and promote the growth and fitness of by inhibiting ethylene signaling	Barazani et al., 2007
*Sesbania sesban*	*Funneliformis mosseae, Rhizophagus intraradicesand Claroideoglomus etunicatum*	Secret the plant hormones	Abd_Allah et al., 2015

### Increase of resistance to stresses for host plants

The references showed that certain endophytic fungi could enhance the resistance of host plants to biotic and abiotic stresses by producing bioactive compounds (chemicals) (Nejad and Johnson, 2000; Cavaglieri et al., 2004) (Table 3). In symbiotically conferred stress tolerance, the endophytic fungi were considered to act as a type of biological trigger that activated the defense systems of a host (Rodriguez and Redman, 2008). For example, endophytic fungi that were inoculated to crop plants improved the resistance and yield of the crops (Kozyrovska et al., 1996), and such a endophytic-mediated plant resistance to pathogens was more likely the result of direct competition between host plants and pathogens.

**Table 3:** **Host medicinal plants with enhanced defense responses conferred by endophytic fungi**.

**Host plant**	**Endophytic fungi**	**Type of stresses**	**Mechanism**	**References**
*Chrysanthemum morifolium*	*Chaetomium globosum, Botrytis* sp.	Salt stress	Increase POD activity and soluble protein content	Liu et al., 2011
*Glycyrrhiza uralensis*	*Arbuseular mycorrhiza, Penicillium griseofulvum*	Drought and salt stress	Reduce injury of water stress by increase pretective enzymes' activity and osmotica contents	Wang L. et al., 2009
*Salvia miltiorrhiza*	*Arbuseular mycorrhiza*	Drought stress	Increase the absorption of nutrient and alter metabolic activities in host	Meng and He, 2011
*Cordia alliodora*	*Leucocoprinus gongylophorus*	Insect	Produce some chemicals antagonistic to ants' fungal symbiont	Bittleston et al., 2011
*Phoenix dactylifera*	*Beauveria bassiana, Lecanicillium dimorphum, L*. cf. *psalliotae*	Insect: date palm pests	Modulate the expression of cell division-related proteins in host	Gómez-Vidal S., mez-Vidal et al., 2009
*Cirsium arvense*	*Chaetomium cochliodes, Cladosporium cladosporioides, Trichoderma viride*	Insect: foliar feeding insects	Produce some chemicals toxic to pathogens	Gange et al., 2012
*Cucumis sativus*	*Chaetomium* Ch1001	Insect: root-knot nematode *Meloidogyne incognita*	Produced abscisic acid affecting motility of the second stage juveniles of insects	Yan et al., 2011
*Picea rubens*	*150 foliar fungal endophytes*	Insects: *Choristoneura fumiferana*	Produce some chemicals toxic to insects	Sumarah et al., 2010
*Atractylodes lancea*	*Gilmaniella* sp. AL12.	Pathogenic fungi	Produce jasmonic acid inducing defense responses	Ren and Dai, 2012
*Curcuma wenyujin*	*Chaetomium globosum* L18	Pathogenic fungi	Produce some chemicals toxic to pathogens	Wang et al., 2012
*Maytenus hookeri*	*Trichothecium roseum*	Pathogenic fungi	Produce trichothecin toxic to pathogens	Zhang et al., 2010
*Phragmites australis*	*Choiromyces aboriginum, Stachybotrys elegans, Cylindrocarpon* sp.	Pathogenic fungi	Produce cell wall-degrading enzymes to kill pathogenic fungi	Cao et al., 2009
*Cassia spectabilis*	*Phomopsis cassiae*	Pathogenic fungi: *Cadosporium sphaerospermum*, and *C. cladosporioides*	Produce cadinane sesquiterpenoids toxic to pathogens	Silva et al., 2006
*Angelica sinensis*	*Bacillus subtilis, Myxormia* sp.	Pathogenic fungi: *Fusarium oxysporum* and *F. Solani*	Produce some chemicals toxic to pathogens	Yang et al., 2012
*Hordeum vulgare* var. *disticum*	*Acremonium blochii, A. furcatum, Aspergillus fumigatus, Cylindrocarpon* sp., *C. destructans, Dactylaria* sp., *Fusarium equiseti, Phoma herbarum, P. leveillei*	Pathogenic fungi: *Gaeumannomyces graminis* var. *Tritici*	Improve the competence for space inhibiting the colonization of pathogens	Maciá-Vicente et al., 2008
*Triticum aestivum* cv. “*Morocco”*	*Chaetomium* sp, *Phoma* sp.	Pathogenic fungi: *Puccinia recondite*	Activate defense reactions of the plant	Dingle and McGee, 2003
*Triptergyium wilfordii*	*Cryptosporiopsis* cf. *quercina*	Pathogenic fungi: *Pyricularia oryzae*	Produce cryptocin and cryptocandin toxic to pathogens	Strobel et al., 1999
*Oryza sativa*	*Sordariomycetes* sp.	Pb^2+^ stress	Inhibition of electron transportfrom the quinone acceptor QA to QB	Li and Zhang, 2015
*Capsicum annuum*	*Penicillium resedanum* LK6	Heat stress	Improve nutrient, proline and flavonoid contents, modulate amino acid metabolism	Khan et al., 2013

Interestingly, in many cases, the tolerance to biotic stresses was correlated with the bioactive compounds produced by endophytic fungi (Saikkonen et al., 1998; Tan and Zou, 2001; Zhang et al., 2006) that had antimicrobial activity against pathogens (Gunatilaka, 2006). Moreover, chemicals produced by endophytic fungi were toxic or distasteful to insects (Hartley and Gange, 2009), protecting the host plants from the attacks of insects. For example, alkaloids produced by endophytic fungi in the genus *Neotyphodium* could confer deterrence to their host plants, increasing their survival from the attacks by insects. With the increased stress tolerance, host plants infected by endophytic fungi could outcompete native plants without fungal infection, and consequently became invasive (Tofern et al., 1999; Clement et al., 2005).

In addition, endophytic fungi could produce a vast variety of antioxidant compounds (Table 3) that could protect their hosts by enhancing tolerance to abiotic stresses (Herrera-Carrillo et al., 2009; Torres et al., 2009). In supporting of this, several studies had demonstrated increased production of antioxidant compounds (e.g., flavonoids and other phenolic antioxidants) in endophyte-infected plants (Richardson et al., 1992; Harper et al., 2003; Huang et al., 2007a,b). Furthermore, it was shown that endophytic fungi possessing metal sequestration or chelation systems were able to increase tolerances of their host plants to the presence of heavy metals, thereby, assisting their hosts to survive in contaminated soil (Weyens et al., 2009).

### Promoting the accumulation of bioactive compounds of medicinal plants

Results from our reference analyses clearly indicated that some endophytic fungi with ability promoted the accumulation of secondary metabolites of host plants, which influenced the quantity and quality of drugs (Chen et al., 2016). Some endophytic fungi could produce diverse classes of phytochemicals—secondary metabolites originally from plants, including the well-known compounds such as paclitaxel (also known as taxol) (Stierle et al., 1993), podophyllotoxin (Eyberger et al., 2006; Puri et al., 2006), deoxypodophyllotoxin (Kusari et al., 2009a), camptothecin, and structural analogs (Puri et al., 2005; Kusari et al., 2009c, 2011; Shweta et al., 2010), hypericin and emodin (Kusari et al., 2008, 2009b), and azadirachtin (Kusari et al., 2012) (Table 4). In fact, the best known example of anticancer compound taxol was found in the taxol-producing endophytic fungi *T. andreanae* that was isolated from *T. brevifolia* (Stierle et al., 1995). Many endophytic fungi colonized in other host plant species, such as S*eimatoantlerium tepuiense, Seimatoantlerium nepalense* (Bashyal, 1999), *Tubercularia* sp. strain TF5 (Wang J. et al., 2006), and *Metarhizium anisopliae* (Liu et al., 2009), were also found to produce taxol.

**Table 4:** **Endophytic fungi producing plant-secondary metabolites in host plants**.

**Endophytic fungi**	**Plant-secondary metabolite**	**Host plant**	**Bioactivity of secondary metabolite**	**References**
*Alternaria* sp.	Berberine	*Phellodendron amurense*	Antibiotic	Duan, 2009
*Fusarium solani*	Camptothecin	*Apodytes dimidiata*	Antitumor	Shweta et al., 2010
*Entrophospora infrequens, Neurospora* sp.	Camptothecin	*Nothapodytes foetida*	Antitumor	Amna et al., 2006; Rehman et al., 2008
*Fusarium solani*	Camptothecin	*Camptotheca acuminata*	Antitumor	Kusari et al., 2009c
*Phomopsis* sp.*, Diaporthe* sp.*, Schizophyllum* sp.*, Penicillium* sp.*, Fomitopsis* sp.*, Arthrinium* sp.	Cinchona alkaloids: quinine, quinidine, cinchonidine, and cinchonine	*Cinchona ledgeriana*	Antipyretic and antimalarial, analgesic and anti-inflammatory	Maehara et al., 2012
*Blastomyces* sp., *Botrytis* sp.	Huperzine A	*Phlegmariurus cryptomerianus*	Anticholinesterase	Ju et al., 2009
*Penicillium chrysogenum*	Huperzine A	*Lycopodium serratum*	Anticholinesterase	Zhou et al., 2009
*Acremonium* sp.*, Shiraia* sp.	Huperzine A,	*Huperzia serrata*	Anticholinesterase	Li et al., 2007
*Cephalosporium corda*	Sipeimine	*Fritillaria ussuriensis*	Antibechic and anti-ulcer	Yin and Chen, 2008
*Alternaria* sp, *Fusarium oxysporum*	Vinblastine	*Catharanthus roseus*	Antitumor	Zhang et al., 1998
*Pestalotiopsis guepinii*	Paclitaxel	*Wollemia nobilis*	Antitumor	Strobel et al., 1997
*Pestalotiopsis terminaliae*	Paclitaxel	*Terminalia arjuna*	Antitumor	Gangadevi and Muthumary, 2009
*Phyllosticta spinarum*	Paclitaxel	*Cupressus* sp.	Antitumor	Senthil Kumaran et al., 2008
*Alternaria* sp.	Paclitaxel	*Ginkgo biloba*	Antitumor	Kim and Ford, 1999
*Phyllosticta dioscoreae*	Paclitaxel	*Hibiscus rosa-sinensis*	Antitumor	Kumaran et al., 2009
*Aspergillus fumigatus*	Paclitaxel	*Podocarpus* sp.	Antitumor	Sun D. et al., 2008
*Phyllosticta citricarpa*	Paclitaxel	*Citrus medica*	Antitumor	Kumaran et al., 2008
*Pestalotiopsis pauciseta*	Paclitaxel	*Cardiospermum helicacabum*	Antitumor	Gangadevi et al., 2008
*Botryodiplodia theobroma, Fusarium lateritium, Monochaetia* sp.*, Pestalotia bicilia*	Paclitaxel	*Taxus baccata*	Antitumor	Venkatachalam et al., 2008
*Taxomyces andreanae*	Paclitaxel	*Taxus brevifolia*	Antitumor	Stierle et al., 1995
*Fusarium solani*	Paclitaxel	*Taxus celebica*	Antitumor	Chakravarthi et al., 2008
*Fusarium solani, Metarhizium anisopliae, Mucor rouxianus*	Paclitaxel	*Taxus chinensis*	Antitumor	Deng et al., 2009; Liu et al., 2009
*Ozonium* sp., *Alternaria alternata, Botrytis* sp., *Ectostroma* sp., *Fusarium mairei, Papulaspora* sp., Tubercularia sp.	Paclitaxel	*Taxus chinensis* var. *mairei*	Antitumor	Zhou et al., 2007; Guo et al., 2009; Wu et al., 2013
*Alternaria* sp., *Aspergillus niger* var. *taxi, Botrytis* sp., *Fusarium arthrosporioide, Pestalotiopsis microspora*	Paclitaxel	*Taxus cuspidata*	Antitumor	Kim and Ford, 1999
*Cladosporium cladosporio*	Paclitaxel	*Taxus media*	Antitumor	Zhang et al., 2009
*Pithomyces* sp.	Paclitaxel	*Taxus sumatrana*	Antitumor	Strobel et al., 1996
*Pestalotiopsis microspora, Sporormia minima, Trichothecium* sp.	Paclitaxel	*Taxus wallachiana*	Antitumor	Shrestha et al., 2001
*Taxomyces* sp.	Paclitaxel	*Taxus yunnanensis*	Antitumor	Qiu et al., 1994
*Periconia* sp.	Paclitaxel	*Torreya grandifolia*	Antitumor	Li et al., 1998
*Pestalotiopsis microspora*	Paclitaxel	*Taxodium distichum*	Antitumor	Li et al., 1996
*Aspergillus nidulans, A. oryzae*	Quercetin	*Ginkgo biloba*	Anti-inflammatory	Qiu et al., 2010
Unidentified	Rutin	*Pteris multifida*	Antibacterial and antioxidant	Fan et al., 2007
*Rhizopus oryzae*	α-Irone, β-Irone	*Iris germanica*	Anti-inflammatory	Zhang L. et al., 1999
*Penicillium implicatum*	Podophyllotoxin	*Diphylleia sinensis*	Antitumor	Zeng et al., 2004
*Monilia* sp., *Penicillium implication*	Podophyllotoxin	*Dysosma veitchii*	Antitumor	Yang et al., 2003
*Penicillium* sp., *Phialocephala fortinii, Trametes hirsuta, Alternaria neesex*	Podophyllotoxin	*Sinopodophyllum hexandrum*	Antitumor	Li, 2007
*Fusarium oxysporum*	Podophyllotoxin	*Juniperus recurva*	Antitumor	Kour et al., 2008
*Alternaria* sp.	Podophyllotoxin	*Sabina vulgaris*	Antitumor	Lu et al., 2006
*Chaetomium globosum*	Hypericin	*Hypericum perforatum*	Anti-depressant	Kusari et al., 2008
*Trichoderma atroviride* D16	Tanshinone IIA and tanshinone I	*Salvia miltiorrhiza*	Antibacterial and anti-inflammatory	Ming et al., 2011
*Sordariomycete* sp.	Chlorogenic acid	*Eucommia ulmoides*	Antimicrobial and antitumor	Chen et al., 2010
*Cephalosporium* sp., *Paecilomyces* sp.	Diosgenin	*Paris polyphylla* var. *yunnanensis*	Antitumor, anti-inflammatory, and cardiovascular-protection	Cao et al., 2007
*Fusarium oxysporum, Neonectria macrodidym, F. solani, F. proliferatum*	Cajaninstilbene acid	*Cajanus cajan*	Antioxidant, hypotriglycerimic, and hypoglycemic	Zhao et al., 2012
*Cochliobolus nisikadoi*	Borneol	*Cinnamomum camphora* chvar. *Borneol*	Anti-inflammatory, antioxidant	Chen M. et al., 2011
*Fusarium oxysporum*	Ginkgolide B	*Ginkgo biloba*	Antishock, antiallergic, and anti-inflammatory	Cui et al., 2012
Unidentified	Toosendanin	*Melia azedarach*	Contact toxicity, stomach toxicity, and anti-feeding	Zhao et al., 2011
*Fusarium redolens*	Peimisine and imperialine-3β-D-glucoside	*Fritillaria unibracteata* var. *wabuensis*	Get rid of sputum, cough, and antitumor	Pan et al., 2015
*Colletotrichum gloeosporioides*	Piperine	*Piper nigrum*	Antimicrobial, antidepressant, anti-inflammatory, and anticancer	Chithra et al., 2014

Other endophytic fungi could promote the formation and accumulation of secondary metabolites that were only produced by host plants. For example, *Coetotrichum gloesporioides* could induce the production of Artemisinin in hairy-root cultures of *Artemisia annua* (Wang J. W. et al., 2006). These compounds commonly function as bioactivities for antitumor, antipyretic, antimalarial, analgesic, or anti-inflammatory in medicinal treatments.

## Conclusion and perspectives

This review highlights the environmental and host-plant factors that can possibly influence the population structure and distribution of endophytic fungi, as well as the benefits these endophtes provide to their host plants.

The fungus-host relationships reveal that the distribution and population structure of endophytic fungi rely largely on the taxonomy, genetic background, age, and tissues of the host plants, in addition to the types of environments. These findings can assist in the investigation of bioactive compounds produced by a certain host medicinal plant under specific environment conditions. In addition, we have observed that there are three types of beneficial interactions between endophytic fungi and their host plants namely: (1) enhancement of the growth of host medicinal plants, (2) increase in the resistance of the host plants to biotic and abiotic stresses, and (3) accumulation of secondary metabolites, including bioactive compounds used as drugs, produced originally by the medicinal plants. These findings have important practical implications for obtaining and producing drugs with improved quality and higher quantity.

Interestingly, genuine medicinal materials with the highest quality and best effects to a certain disease seems to have a special relationship with endophytic fungi. Special types of endophytic fungi of medicinal plants may be associated with the production of specific bioactive compounds needed by human. For example, a medicinal plant *Huperzia serrata* found in tropical region can produce Huperzine-A compounds that are considered being stimulated by endophytic fungi *Acremonium* sp. and *Shiraia* sp. (Wang Y. et al., 2009; Wang et al., 2011; Zhou et al., 2009). This is the reason why in traditional Chinese medicine, doctors prefer to use a particular medicinal plants from a particular geographical locations or habitats where the content and chemical types of particular compounds can be expected. Therefore, understanding the distribution and population structure patterns of endophytic fungi will provide a theoretical guide for effectively exploring bioactive compounds of drugs produced by a special host medicinal plant in particular tissues under special environment conditions.

Importantly, the application of target endophytic fungi can promote seed germination of many host plant species. The significance of this application can increase opportunities for the germination of those seeds that cannot germinate under the normal conditions. For example, seeds of some rare and endangered medicinal species, such as *Dendrobium nobile* and *D*. *chrysanthum* in the orchid family, are extremely difficult to geminate under normal conditions However, with the application of endophytic fungi in the genus *Mycena*, these seeds can germinate successfully, which has facilitated the artificial culture of these medicinal plants (Chen and Guo, 2005). This is particularly useful for the rare and endangered medicinal plants that are used in breeding programs where seed germination is crucial.

The most valuable application is to utilize the advantages of endophytic fungi that can promote the accumulation of secondary metabolites originally produced by plants. Through such an application, we can enhance the synthesis and accumulation of bioactive compounds of the host medicinal plants for higher quality of crude drugs, by adding particular endophytic fungi to the plants. This application may open a complete new dimension for the production of natural medicines in an extremely effective manner, given that the relationship between endophytic fungi and their host medicinal plants is completely understood.

Unfortunately, much of the work reported on the beneficial strains is confined to experimental studies, and more efforts should be put into field trials and applications to obtain higher-quality drugs. Also, the mechanisms of the interactions between endophytic fungi and their host plants have not been clearly defined. In addition, the research emphasis of endophyte need to be addressed over the next several decades, such as:

- Build a bioengineering system to mimick the mutualistic/antagonism symbiosis of endophytic fungi and their host plants, and facilitate the production of the bioactive compounds.
- Set up a guide for rapid screening of plant endophytic fungi beneficial to host plants other than isolate all strains uncritically.
- Establish target endophytic fungi library for plant breeding in order to protect the endangered medicinal plants by using seed germination.
- Solve the degradation problem of target endophytic fungi that can produce desired metabolites.
- Make better use of beneficial strains in planting and cultivating medicinal plants so the pharmaceutical products can be improved.

Such knowledge can be well exploited and applied for obtaining better drugs from medicinal plants. We believe that this review provides new insights into drug discovery and clinical utility which can be further improved by investigating endophytes further as these have the potential of playing a key front line role in the treatment of various diseases.

## Author contributions

Reviewed and finalized manuscript: TH, LQ; Completed the article writing: MJ, LC; Integrated information of tables, analyzed data, and made pictures: HX, CZ; Took charge of the manuscript language: KR.

### Conflict of interest statement

The authors declare that the research was conducted in the absence of any commercial or financial relationships that could be construed as a potential conflict of interest.
